# High STAT1 mRNA levels but not its tyrosine phosphorylation are associated with macrophage infiltration and bad prognosis in breast cancer

**DOI:** 10.1186/1471-2407-14-257

**Published:** 2014-04-12

**Authors:** Piotr Tymoszuk, Pornpimol Charoentong, Hubert Hackl, Rita Spilka, Elisabeth Müller-Holzner, Zlatko Trajanoski, Peter Obrist, Françoise Revillion, Jean-Philippe Peyrat, Heidi Fiegl, Wolfgang Doppler

**Affiliations:** 1 Division of Medical Biochemistry, Biocenter, Innsbruck Medical University, Innrain 80-82, 6020 Innsbruck, Austria; 2 Division of Bioinformatics, Biocenter, Innsbruck Medical University, Innsbruck, Austria; 3 Laboratory of Pathology, Hospital St. Vinzenz, Zams, Austria; 4 Department of Obstetrics and Gynecology, Innsbruck Medical University, Innsbruck, Austria; 5 Centre Oscar Lambret, Lille Cedex, France

## Abstract

**Background:**

STAT1 has been attributed a function as tumor suppressor. However, in breast cancer data from microarray analysis indicated a predictive value of high mRNA expression levels of STAT1 and STAT1 target genes belonging to the interferon-related signature for a poor response to therapy. To clarify this issue we have determined STAT1 expression levels and activation by different methods, and investigated their association with tumor infiltration by immune cells. Additionally, we evaluated the interrelationship of these parameters and their significance for predicting disease outcome.

**Methods:**

Expression of *STAT1*, its target genes *SOCS1*, *IRF1*, *CXCL9*, *CXCL10*, *CXCL11*, *IFIT1*, *IFITM1*, *MX1* and genes characteristic for immune cell infiltration (*CD68*, *CD163*, *PD-L1*, *PD-L2*, *PD-1*, *CD45*, *IFN-γ*, *FOXP3*) was determined by RT-PCR in two independent cohorts comprising 132 breast cancer patients. For a subset of patients, protein levels of total as well as serine and tyrosine-phosphorylated STAT1 were ascertained by immunohistochemistry or immunoblotting and protein levels of CXCL10 by ELISA.

**Results:**

mRNA expression levels of STAT1 and STAT1 target genes, as well as protein levels of total and serine-phosphorylated STAT1 correlated with each other in neoplastic tissue. However, there was no association between tumor levels of STAT1 mRNA and tyrosine-phosphorylated STAT1 and between CXCL10 serum levels and CXCL10 expression in the tumor. Tumors with increased STAT1 mRNA amounts exhibited elevated expression of genes characteristic for tumor-associated macrophages and immunosuppressive T lymphocytes. Survival analysis revealed an association of high STAT1 mRNA levels and bad prognosis in both cohorts. A similar prognostically relevant correlation with unfavorable outcome was evident for CXCL10, MX1, CD68, CD163, IFN-γ, and PD-L2 expression in at least one collective. By contrast, activation of STAT1 as assessed by the level of STAT1-Y701 phosphorylation was linked to positive outcome. In multivariate Cox regression, the predictive power of STAT1 mRNA expression was lost when including expression of CXCL10, MX1 and CD68 as confounders.

**Conclusions:**

Our study confirms distinct prognostic relevance of *STAT1* expression levels and STAT1 tyrosine phosphorylation in breast cancer patients and identifies an association of high STAT1 levels with elevated expression of STAT1 target genes and markers for infiltrating immune cells.

## Background

STAT1 has been described as a tumor suppressor because of its function as a mediator of IFN-γ - dependent immunosurveillance [1]. This anti-tumor activity of STAT1 appears to be particularly important at the onset of tumor formation and is supposed to lead to the elimination of transformed cells by the innate and adaptive immune system. At a cellular level, STAT1 can exert this function via shaping the immune effector phenotype [2,3]: In dendritic cells, genes required for antigen processing and presentation are up-regulated, and in macrophages cytotoxic activity is increased, e.g. by induction of *iNOS* expression. STAT1 also participates in the differentiation of B cells [4,5] and T cells [6,7]. In addition to its function in immune cells, expression of *STAT1* in the tumor epithelium has been shown to exert an inhibitory effect on the development of the tumor [8,9]. This has been attributed to its cell-autonomous role in mediating apoptosis and proliferation arrest in response to cellular stress such as oncogenic transformation [10], as well as to the transcriptional induction of chemokine and MHC class I genes, which promote recruitment of immune effector cells and recognition of tumor antigens [2].

The proof of principle for the importance of STAT1 in impeding the development of tumors came from experiments with MMTV-neu tumor STAT1 null mice, which develop mammary tumors with shorter latency as compared to STAT1-proficient controls [8,9,11]. Furthermore, STAT1 deficiency predisposed multiparous wild-type mice to intraepithelial neoplasias [12]. Expression of *STAT1* in the tumor epithelium as well as in the stroma cells was shown to contribute to these anti-tumor effects of STAT1 [8,9,12,13]. It has been postulated from these observations that tumors may adapt to the anti-tumor action of STAT1 by down-regulating its expression and/or by impairing its activation [1]. This notion is supported by immunohistochemical (IHC) analysis of *STAT1* expression in estrogen receptor (ER) - positive primary mammary carcinomas, which revealed lower *STAT1* expression levels in the tumor epithelium as compared to the adjacent normal epithelium in a considerable number of cases [14].

In addition to these effects of STAT1 in preventing development and progression of early lesions an influence of STAT1 on the progression of established tumors and their response to therapy has been described. Forced over-expression of *STAT1* in tumor cells was found to confer resistance to radiotherapy [15] and tumor cells with an increased propensity to metastasize to the lung after serial transplantations were shown to acquire a phenotype characterized by high expression levels of *STAT1 *[16]. Furthermore, increased expression of genes belonging to the so-called interferon-related gene signature including *STAT1* was shown to correlate with elevated frequency of relapse in human breast cancer [17]. Moreover, the presence of tumor STAT1 activity correlated with disease progression from ductal carcinoma in situ to invasive carcinoma [18]. It has been proposed that high expression of *STAT1* in established tumors could be the result of a selection process and promote the escape of tumor cells from IFN-γ-mediated tumor surveillance [19]. On the other hand, activation of STAT1 in established mammary tumors as determined by specific DNA binding activity and tyrosine phosphorylation was linked to good prognosis and decreased frequency of disease recurrence [20], indicating that high expression levels and activation of STAT1 might represent distinct prognostic and /or predictive parameters.

Alternatively to its potential direct impact on tumor progression, STAT1 expression and activation might serve as markers for chronic or acute inflammatory processes in the tumor, which are known to potentially influence the progress of disease depending on the type of infiltrating cells and tumor subtypes [21,22]: this is because IFNs, the major triggers of STAT1 expression and activation in the tumor epithelium and stroma, are secreted during acute as well as chronic inflammatory responses [23]. In order to better understand the interrelationship between STAT1 expression and activation, progression of disease and immune infiltration, the expression of *STAT1* and STAT1 target genes as well as marker genes for infiltrating immune cells was analyzed in primary mammary carcinoma tissue derived from two independent patient cohorts. The data were evaluated by correlation analysis for a link between STAT1 and immune infiltrates as well as for their significance in predicting progression of disease and patient’s survival. The study revealed a link of potential mechanistic significance between elevated expression of *STAT1* and its target genes with markers of infiltrating immune cells, in particular with tumor-associated macrophages.

## Methods

### Patients

Two patient cohorts are included in this retrospective study: Cohort A represents 96 breast cancer patients who underwent surgery at the Department of Gynecology and Obstetrics, Innsbruck Medical University between 1989 and 2003; cohort B comprises 36 patients treated at the Oscar Lambret Anticancer Center of the North of France, Lille. Clinical and pathological characteristics of the two populations are summarized in Table 1. The distribution of clinical features in these cohorts was representative for patients treated at the respective clinical centers. 54% of the patients in cohort A and 44% of the patients in cohort B were diagnosed with ER-positive infiltrating ductal cancer. For both cohorts mRNA prepared from the primary tumor was available. From cohort A, paraffin blocks for IHC analysis and additional material for immunoblotting as well as serum for ELISA analysis were provided for the study. The number of patient samples available for each type of analysis performed in this study is specified in Additional file 1. Breast cancer samples from patients undergoing surgery for locoregional disease were obtained in accordance with Austrian law and principles of Declaration of Helsinki with the agreement of the Ethics Committee of the Innsbruck Medical University (reference number: UN4051) and the investigator‘s Institutional Review Board in the Centre Oscar Lambret, Lille. Due to the retrospective nature of our study it was not possible to obtain written consent from every patient. The need for patient consent was therefore waived for these cases by the institutional review board. All samples were anonymized to guarantee the protection of privacy before performing the analysis.

**Table 1:** Characteristics of breast cancer patients and tumors

	**Cohort A**		**Cohort B**	
Characteristic		% of patients		% of patients
Total patients, n	96		36	
Age at diagnosis, years				
Mean	58		59	
Range	30-90		35-80	
Menopause, n	68	71	-	
Histotype, n				
Infiltrating ductal cancer	73	76	21	58
Infiltrating lobular cancer	10	10	1	3
Others	13^a^	14	14	39
Stage, n				
I	34	35	1	3
II	55	57	20	56
III/IV	7	7	11	31
Unknown	0		4	10
Lymph node status, n				
Positive	54	56	19	53
Negative	38	40	17	47
Unknown	4	4	0	
ER status, n				
Positive	67	70	23	64
Negative	26	27	13	36
Unknown	3	3	0	
Tumor recurrence, n	31	32	19	53
Patient death, n	52	53	14	39
Follow-up, years				
Median	9.1		6.8	
Range	0.1-22		0.6-10	

^a^three mucinous, four medullary, one comedo, five unknown.

### Immunohistochemical detection of STAT1

After deparaffinization and rehydration, 3 to 5 μm thick tissue sections were placed for 10 min in 10 mM citrate buffer (pH 6.0) at 95°C for antigen unmasking, followed by treatment with 3% H_2_O_2_ for 10 min at room temperature to block endogenous peroxidase. Staining for STAT1 was performed with the Vectastain ABC Kit (Vector Laboratories, Burlingame, CA) using a 1:250 dilution of primary STAT1 rabbit monoclonal antibody (42H3, Cell Signaling) in SignalStain® Antibody Diluent (Cell Signaling) and overnight incubation at 4°C. Slides were treated with the chromogen diaminobenzidine and counterstained with Mayer’s Hemalum (Merck).

### Evaluation of slides

Antigen expression was defined as the presence of specific staining in the cytoplasm or nucleus of cells. The proportion of stained tumor or stroma cells (0, none; 1, <10%; 2, 10-50%; 3, >50%) as well as the intensity of staining (0, none; 1, weak; 2, moderate; 3, strong) was independently evaluated by two examiners and slides revisited in the case of differences in scoring. The total immunostaining score was calculated individually for tumor and stroma cells and was defined as the product of proportion and intensity score (range 0 to 9). Histograms with the number of cases for each score are shown in Additional file 2.

### Protein extracts and immunoblotting

Whole cell extracts were prepared from tumor material and normal adjacent tissue pulverized under liquid nitrogen as described previously [20]. Samples were run on SDS-PAGE gels, proteins transferred to poly(vinylidene) difluoride membranes and probed with primary antibodies for STAT1 (#9176, 1:1000, Cell Signaling), pS727-STAT1 (#07-307, 1:1000, Millipore) and pY701-STAT1 (#9171, 1:500, Cell Signaling). For immunodetection, the enhanced chemiluminescence protocol of Amersham (GE Healthcare) was used. Quantification of the abundance of the immunoreactive STAT1 specific band and normalization of different experiments was performed as described [20].

### CXCL10 ELISA

The protein concentration of CXCL10 in serum samples or whole cell extracts from pulverized tumor tissue were determined with a commercial ELISA set (Human IP-10 ELISA development Kit, PeproTech). Whole cell extracts were prepared as described [20].

### RNA preparation and RT-PCR

RNA from pulverized tumor and adjacent tissue was prepared as described [24], reverse transcribed and analyzed by quantitative RT-PCR as reported previously [25] using the expression of TATA-box binding protein (*TBP*) as a reference for normalization. In all cases, the efficiency of amplification by the designed primers was more than 80% as determined by serial dilutions of the standard. Amplification of the gene products was performed essentially as described with either the TaqMan [26] or EvaGreen methodology [11]. Reactions included two standard control tumor cDNA samples and a non-template control. Expression levels are represented as relative amount of specific cDNA versus TBP using the Delta Ct method and the formula 2^Ct(TBP)-Ct(gene)^. Delta Ct values were used for the correlation studies. For primer and probe sequences, see Additional file 3. For analysis of CD45 expression the primers were obtained from Applied Biosystems.

### Statistical analysis

For the association of *STAT1* expression with other gene expression data, Spearman’s and Pearson‘s correlation coefficients and their two-tailed significances were determined as appropriate. For survival analysis, patients were categorized into two groups using the median of the delta Ct expression value. Overall survival (OS) was taken as time from the initial tumor resection to death, and relapse free survival (RFS) as the time from tumor resection to date of recurrence. Survival curves were generated using the Kaplan–Meier method and compared using the log-rank (Mantel-Cox) test. Hazard ratios (HR) with their corresponding 95% confidence intervals (CI) were estimated using Cox proportional hazards models. Levels of significance are indicated by stars: *, p value < 0.05; **, p value < 0.01; ***, p value < 0.001. Analyses were performed using SPSS and R platform software. Heatmaps were generated using Genesis [27].

## Results

### STAT1 protein levels in the tumor correlate with STAT1 mRNA levels

**Figure 1:**
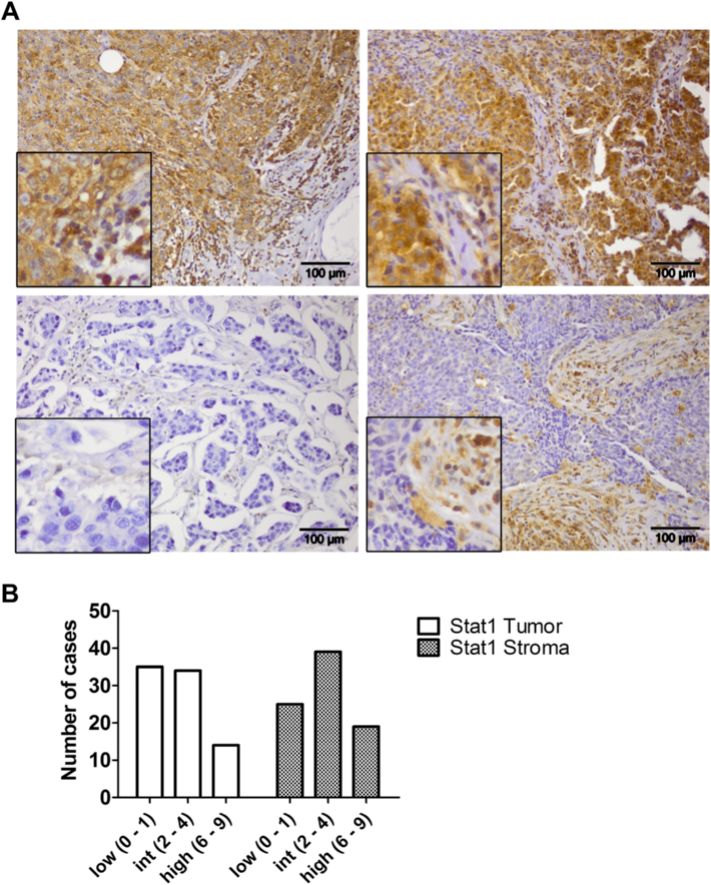
**STAT1 specific IHC staining of human primary mammary tumors.** Sections of paraffin embedded material from 83 primary mammary tumors were investigated. **(A)** Examples for tumors with strong staining of tumor epithelium and stroma (upper left), predominant staining of tumor epithelium (upper right), weak staining (lower left), and strong staining specific for stoma (lower right). **(B)** Histograms for STAT1 total immunostaining score in tumor epithelium or stroma.

**Figure 2:**
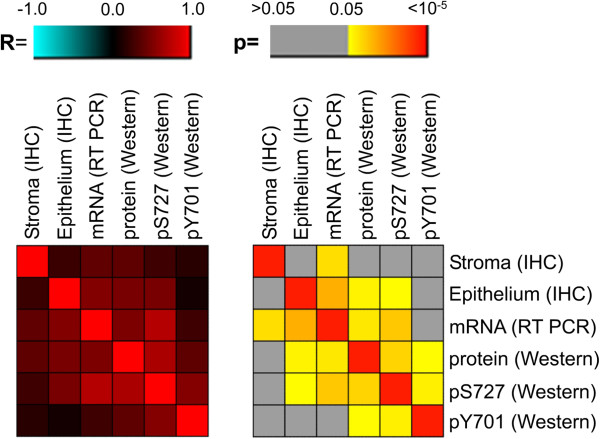
**Comparison of *STAT1 *expression levels as determined by IHC, RT-PCR and immunoblotting.** Total STAT1 immunostaining scores for either stroma or tumor epithelium obtained with paraffin-embedded material were correlated with mRNA expression and protein expression levels in tumor extracts. Both total STAT1 as well serine (S727) or tyrosine (Y701) phosphorylated STAT1 was determined by Western blotting. Correlation coefficients (R) and significance levels (p) were calculated by Spearman’s rank correlation and are represented by color coding.

STAT1 protein levels in tumor epithelium and stroma were separately assessed by immunohistochemical staining of primary breast cancer tissue derived from 83 patients. Typical staining patterns are shown in Figure 1A. Expression was found to be highly variable: Some tumors exhibited strong staining both in the stroma and tumor epithelium, whereas in others either the epithelium or stroma was predominantly stained. 42% and 30% of the tumors exhibited no or weak staining for STAT1 in the epithelium or stroma, respectively (Figure 1B). Pulverized tumor tissue obtained from the same patients was analyzed for STAT1 mRNA and protein by RT-PCR and western blotting. Expression levels determined by immunohistochemistry (IHC) in the tumor epithelium correlated significantly with the values obtained by RT-PCR and western blotting (Figure 2), thus validating the specificity of the different detection methods. STAT1 protein in the stroma correlated with mRNA expression levels only, pointing to a predominant influence of tumor epithelium-expressed *STAT1* on the total measured protein levels in extracts of pulverized tissue. Interestingly, the activation of STAT1 as assessed by immunoblotting with the pS727-specific antibody but not with the Y701-specific antibody was correlated with total STAT1 protein or mRNA levels as determined by IHC or RT-PCR (Figure 2). Thus activation hallmarked by phospho-serine 727 does not appear to be coupled to tyrosine 701 phosphorylation in the set of 27 investigated tumors.

### No evidence for downregulation/mutation of *STAT1* expression as driving force for the expansion of human mammary tumors

**Figure 3:**
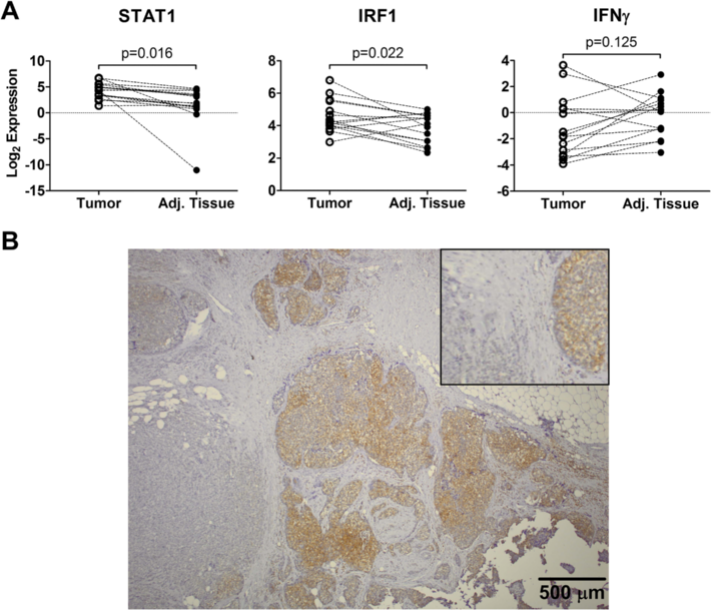
***STAT1 *expression in tumor vs. tumor free adjacent tissue and in different areas of the same tumor. (A)** mRNA expression levels of *STAT1* and the STAT1 target gene *IRF1* in tumor areas and matched tumor-free adjacent tissue were determined in 15 pairs of tissue. Statistical significance was determined with paired Student’s T-test. **(B)** Example for a tumor with strong and weak staining for STAT1 in neighboring regions.

The entire coding region of STAT1 cDNA derived from tumor mRNA of seven ER-positive and two ER-negative mammary tumors exhibiting lowered levels of STAT1 protein was sequenced. Tumor epithelium content in these samples was more than 90% as checked by histological analysis of frozen sections from this area. No mutations were detectable, indicating that at least in these cases, mammary tumors did not expand because of a mutation in *STAT1*. We have further analyzed mRNA expression of *STAT1* and its target gene *IRF1* in paired samples of tumor and adjacent tumor-free tissue and found a significant increase in expression of both genes in neoplasm rather than a decrease (Figure 3A). Notably, expression of *IFN-*γ was similar in the tumor and adjacent tissue (Figure 3A), indicating that a higher production of IFN-γ by immune cells in the tumor did not contribute to the increased mRNA levels of STAT1 and IRF-1.We cannot rule out that the increased levels of STAT1 mRNA STAT1 and IRF-1 in the tumor are the result of higher abundance of immune cells expressing these genes in the tumor.

Another argument against the downregulation of *STAT1* as a driving force for tumor development comes from the observation that some of the tumors contained areas with high *STAT1* expression adjacent to areas with no detectable *STAT1* expression, without an apparent difference in the histological grading between these areas. An example for a such tumor is shown in Figure 3B. If STAT1-deficiency conferred a competitive advantage to the tumor cells in the developing tumor, a selective expansion of STAT1-negative areas would be expected. This was however not the case in any of the evaluated samples.

### Coordinate regulation of *STAT1*, STAT1 target genes and markers for tumor infiltration with leukocytes

**Figure 4:**
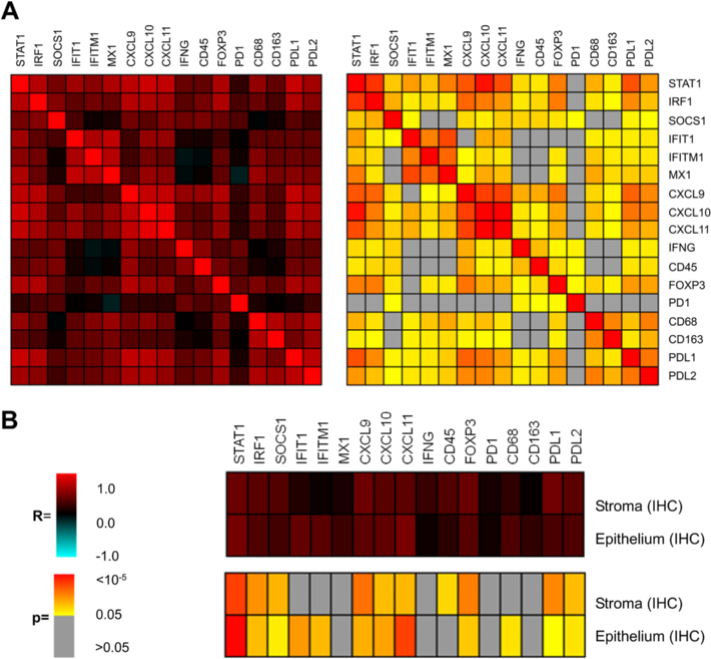
**Correlation of mRNA levels of STAT1, STAT1 target genes and lymphocytes and macrophage-specific genes.** mRNA expression levels determined by quantitative RT-PCR in primary tumors of cohort A were compared in the correlation. IRF1, a transcription factor proposed as a mediator of the anti-tumor action of STAT1; SOCS1, a feed back inhibitor of STAT1 signaling; the antiviral proteins IFIT1, IFITM1 and MX1; and the chemokines CXCL9, CXCL10 and CXCL11 involved in the recruitment of immune cells, in particular T cells, are encoded by STAT1 target genes. CD45 is a pan-leukocyte marker highly expressed in lymphocytes. FOXP3, PD-1 are predominantly expressed in T cells. IFN-γ is expressed by activated T cells, by NK cells, NK T cells and professional antigen presenting cells. PD-L1 is induced by IFN-γ in different cell types including macrophages. PD-L2 is specifically expressed in macrophages and dendritic cells. CD68 and CD163 represent markers for macrophages. **(A)** Correlation matrix. **(B)** Correlation with STAT1 IHC staining scores in tumor epithelium and stroma. Correlation coefficients (R) and significance levels (p) were determined by Pearson’s correlation and are represented by color coding.

**Figure 5:**
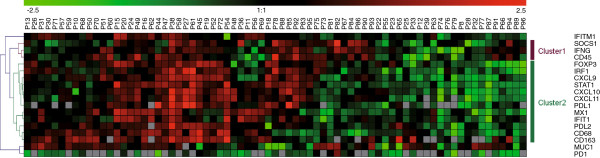
**Clustering of patients according to gene expression patterns of *STAT1*, STAT1 target genes and genes expressed in lymphocytes and macrophages.** Mammary tumor expression data from 74 patients of cohort A were normalized and hierarchically clustered (Pearson uncentered, distance measure, average linkage algorithm). Grayed squares indicate not available data.

mRNA expression analysis of *STAT1*, STAT1 target genes and markers for infiltrating lymphocytes and macrophages was performed by RT-PCR. The obtained values were positively associated with each other (Figure 4A), with the exception of PD-1. Among the investigated genes, an especially high inter-correlation of expression values was observed within subgroups encoding proteins with similar function or those serving as markers for tumor-infiltrating immune cells: subgroup a, antiviral proteins IFIT1, IFITM1, MX1; subgroup b, chemokines CXCL9, CXCL10, CXCL10; subgroup c, immune cell markers IFN-γ, CD45, FOXP3, subgroup d, macrophage marker proteins CD68, CD163; and subgroup e, immunosuppression-related proteins FOXP3, PD-L1, PD-L2. Of note, in addition to the well characterized role in a subset of regulatory T-cells, FOXP3 was reported to be functional also in mammary epithelial cells as a breast cancer suppressor gene [28]. However, in human breast cancer, nuclear expression levels of FOXP3 were found to be negligible in comparison to FOXP3-positive T-cells [29]. PD-L2, originally detected in macrophages and dendritic cells [30,31], was later also described to be induced in other types of immune and non – immune cells [32]. The expression of antiviral genes IFIT1 and IFITM and macrophage-marker CD68 was found to correlate exclusively with the epithelial STAT1, whereas for the pan-leucocyte marker *CD45* significant correlations were restricted to the stroma. (Figure 4B). The other genes exhibited no selective association with expression of STAT1 in one of the tumor compartments. In a hierarchical clustering analysis, the panel of genes investigated in Figure 4 formed two major clusters (Figure 5). Cluster 1 comprised *CD45*, *IFN-*γ and *SOCS1*. Cluster 2 contained *STAT1*, its target genes *IRF1*, *CXCL9*, *CXCL10* and *CXCL11* as well as macrophage marker and immunosuppression-related genes. In conclusion, correlation and clustering analysis revealed an interrelationship between markers for macrophages, immunosuppression and STAT1 transcriptional activity. Interestingly, expression of the macrophage marker CD68 was specifically associated with tumor epithelium-specific *STAT1* expression.

### Independent regulation of serum and tumor levels of the STAT1 target gene *CXCL10*

The coordinate upregulation of STAT1 target genes and markers for immune infiltration indicates a close interplay between activity of the immune system and induction of STAT1 target genes in mammary tumors. The question arises whether this interaction is restricted to the tumor or may also reflect a systemic response which can be monitored by analysis of serum samples of patients for elevated levels of proteins induced by STAT1. *CXCL10* represents a suitable STAT1 target gene for such analysis [2]. It is secreted by cells of inflamed tissues in response to IFN-γ. Due to its relatively long half live in the serum it can serve as a sensitive readout of activation of the innate immune system in course of/during various diseases [33-37]. We analyzed the concentration of CXCL10 in preoperative sera and tumor samples of 10 patients and compared these values with the expression levels of CXCL10 and STAT1 mRNA in the tumor (Table 2). The experiments revealed a stringent association of protein and mRNA expression levels of *CXCL10* and *STAT1* in the tumor. However, there was no association between CXCL10 protein levels in serum and tumor tissue, indicating that STAT1 activity in the tumor compartment cannot be simply predicted by monitoring serum levels of CXCL10.

**Table 2:** Correlation of CXCL10 levels in serum with CXCL10 and STAT1 expression levels in the tumor of 10 breast cancer patients

		**CXCL10 tumor (pg/mg protein)**^**a**^	**CXCL10 tumor (mRNA levels)**^**c**^	**STAT1 tumor (mRNA levels)**^**c**^
CXCL10 serum (pg/ml)^a^	r^d^	0.374	0.497	0.460
	p-value^e^	0.287	0.144	0.181
CXCL10 tumor (pg/mg protein)^b^	r^d^		0.915	0.636
	p-value^e^		0.0002	0.048
CXCL10 tumor (mRNA levels)^c^	r^d^			0.745
	p-value^e^			0.013

^a^CXCL10 levels in the serum of breast cancer patients collected at time of diagnosis prior to surgery.

^b^CXCL10 levels in extracts of the primary tumor.

^c^relative mRNA expression normalized to the expression of TBP as determined by RT-PCR.

^d^Spearman‘s rho correlation coefficient.

^e^two-tailed significance of Spearman‘s rho.

### mRNA levels of STAT1 and markers for macrophage infiltration are predictive for bad prognosis in breast cancer, whereas STAT1 tyrosine phosphorylation is associated with favorable outcome

**Figure 6:**
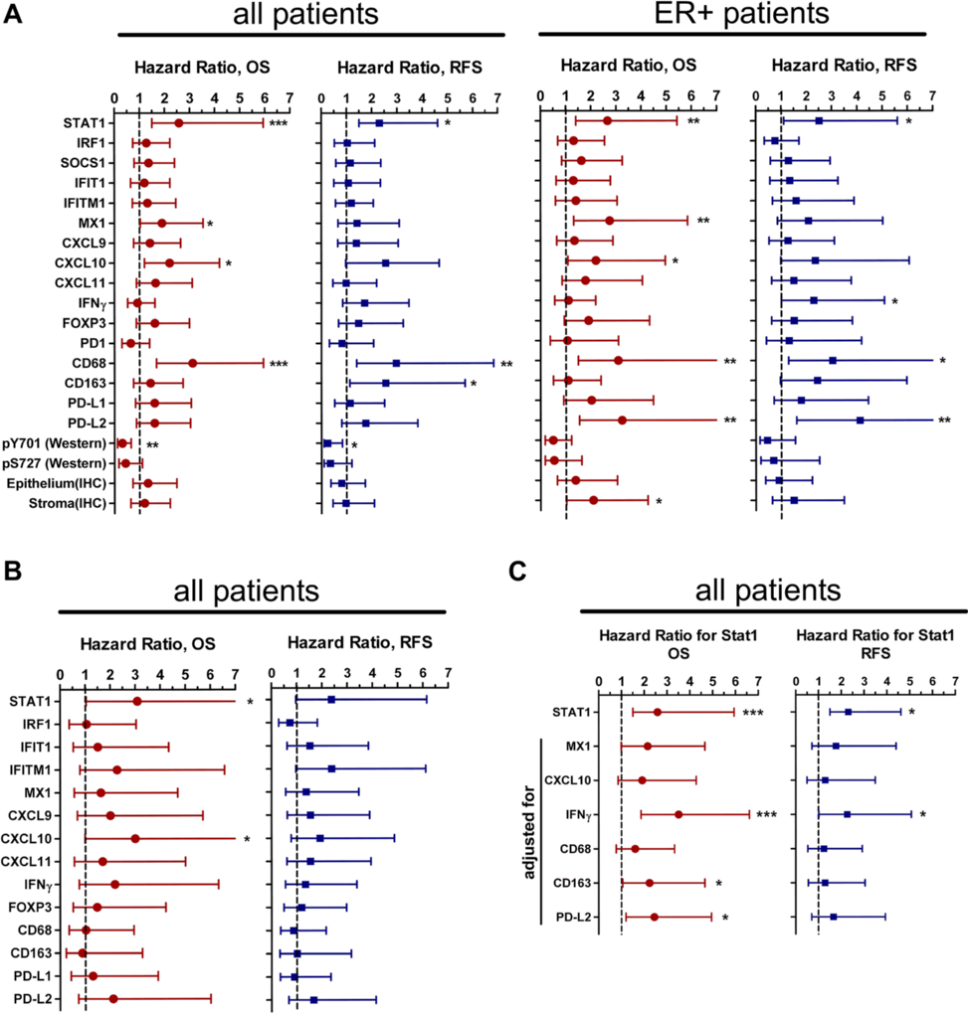
**Prognostic value of gene expression in primary mammary tumors related to STAT1 and leukocyte infiltration.** mRNA expression levels of *STAT1*, STAT1 target genes, genes expressed in lymphocytes and macrophages, as well as STAT1 Y701 phosphorylation and STAT1 IHC scores were used to categorize patients into two groups with the median as discriminator. Hazard ratios for overall survival (OS) and relapse-free survival (RFS) were evaluated by Cox regression for patients with high vs. low expression and are shown as mean together with the 95% confidence interval. **(A)** Cohort A, all patients (n = 96) and the subset of ER-positive patients (n = 67) were evaluated; **(B)** Cohort B (n = 36). **(C)** Cohort A, all patients (n = 96), multivariate Cox regression analysis for patients with high vs. low STAT1 mRNA expression adjusted to expression of genes found to be significantly linked to bad prognosis in the evaluation of Panel **A**. Number of tumor samples with available data for expression analysis for the different genes in the two different cohorts are shown in Additional file 1.

**Figure 7:**
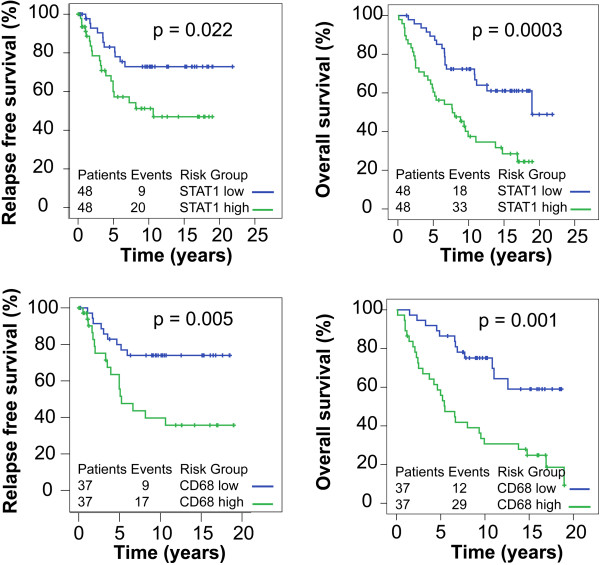
**Kaplan-Meier plots for STAT1 and CD68 high and low groups (OS and RFS).** Kaplan-Meier curves for overall survival (OS) and relapse-free survival (RFS) of patients in cohort A with high vs. low mRNA expression levels in their primary tumor are shown. P-values for significance of difference between high and low expression were calculated the by the log-rank test.

The impact of *STAT1*, its target genes and markers for leukocyte infiltration on patient’s prognosis was examined. Hazard ratios for overall survival (OS) and relapse-free survival (RFS) were calculated for patients with high vs. low mRNA expression of these genes in two independent cohorts (cohort A and B) comprising a total of 132 individuals (Figure 6A and B). In cohort A the analysis revealed a significantly increased risk of death and recurrence for patients with high STAT1 and CD68. This was also evident from the corresponding Kaplan-Meier plots shown in Figure 7. mRNA levels of MX1, CXCL10, CD163, and PD-L2 were significantly associated with either high hazard ratio in at least one of the four evaluations shown in Figure 6A (OS or RFS, all patients or ER-positive patients of cohort A). In cohort B the link of high STAT1 and CXCL10 expression with bad prognosis was also apparent (Figure 6B). Among macrophage markers, only PD-L2 tended to associate with heightened mortality and recurrence rate in this collective.

The prognostic value of epithelial and stromal STAT1 protein levels as well as S727 and Y701 STAT1 protein phosphorylation was assessed in a subset of cohort A (Figure 6A). STAT1 protein levels determined by IHC were less effective at predicting disease outcome than STAT1 mRNA. A significantly increased mortality risk was observed only for the stroma IHC score in the ER-positive subset of patients. As reported previously [20], the elevated levels of STAT1 pY701 were predictive of favorable disease outcome. STAT1 tyrosine phosphorylation therefore appears to reflect a different state of STAT1 activation as also indicated by the lack of its correlation with STAT1 mRNA levels (Figure 2). Similarly, high STAT1 pS727 levels could be linked to the lowered risk. This association was however not significant, possibly due to the small number of analyzed individuals (Figure 6A and B).

In a multivariate Cox regression analysis performed with all patients of cohort A, the predictive power of STAT1 was lost or reduced when including the expression of either MX1, CXCL10, CD68, CD163 or PD-L2 as confounders (Figure 6C). This indicates that these genes are not only linked to STAT1 by concomitant regulation of their expression as shown in Figure 4, but may also contribute to the prognostic significance of STAT1.

## Discussion

The most striking result of our study is the opposing impact of STAT1 pY701 levels versus STAT1 expression and transcription of STAT1 target genes on prognosis in mammary cancer. A similar distinct association of STAT1 and pY-STAT1 levels with patient’s survival has been recently reported for soft tissue sarcomas [38]. Mechanistically, our findings can be potentially attributed to different outcomes of short-term and prolonged STAT1 signaling in the tumor. STAT1 Y701 phosphorylation is typically maximal within the first hour of extracellular stimulation of JAK/STAT signaling, e.g. by IFN-γ, and then decreases as a result of the action of counter-regulatory phosphatases [39,40] and the negative feedback elicited by SOCS1 [41,42]. On the other hand, STAT1 transcriptional activity usually remains elevated even after cessation of triggering signals and leads to a sustained upregulation of STAT1 target genes. Long-term effects of STAT1 activation are further enhanced by upregulation of STAT1, which stays under its own transcriptional control, and is described to act as a transcription factor even in unphosphorylated form [43,44]. Thus, the increased levels of pY-STAT1 and its association with good prognosis in breast cancer tissue may reflect the short-term mode of STAT1 signaling (Figure 6A), whereas elevated STAT1 and STAT1 target gene mRNA and its link with bad prognosis may be indicative of persistent stimulation of Jak/STAT1 signaling in malignant cells (Figure 6A and B). Furthermore, the postulated disparity in kinetics of STAT1 signaling in breast cancer tumors may underlie the lack of linkage between pY-STAT1 levels and STAT1 mRNA expression (Figure 2).

Another important posttranslational modification of STAT1 is the phosphorylation at S727 [45]. pS727-STAT1 was reported to stimulate or inhibit the IFN-γ transcriptional response depending on the target gene [46]. S727 phosphorylation usually follows after Y701 phosphorylation [45]. However, there are number of reports describing a separate regulation of these two sites. For example, in NK cells the phosphorylation of S727 can occur without concomitant tyrosine phosphorylation [47] and in macrophages adenosine A(3) receptor signaling selectively modulates S727 phosphorylation [48]. Our findings on the lack of correlation of pS727-STAT1 and pY701-STAT1 levels in breast cancer tissue samples (Figure 2) provide a further example for the non-coordinate regulation of these two sites. In our study we could observe an apparent discrepancy, whereby the total STAT1 protein levels as determined by IHC or the pS-727 STAT1 levels as quantified by immunoblotting were not significantly correlated with bad prognosis (Figure 6A), despite being linked to STAT1 mRNA expression levels (Figure 2). The STAT1 protein, however, could be regulated at the levels of transcription and translation. Since only the *STAT1* mRNA levels were found to be predictive of unfavorable outcome, we postulate that only the transcriptional but not the posttranscriptional regulation is relevant for the prognosis. This implicates that the protein may serve as a rather unspecific readout of STAT1 transcriptional activity.

How STAT1 is activated in mammary tumors remains unclear. Its activation might be promoted by tumor-intrinsic mechanisms mediated by receptor tyrosine kinases, such as HER2/erbB2 [9] or induced by its principle activator IFN-γ produced by immune cells. The latter possibility is supported by the association of IFN-γ and marker genes for infiltrating immune cells in tumors with high STAT1 levels (Figure 4). Elevated levels of IFN-γ in ER-positive tumors were predictive of bad prognosis, as were high STAT1 levels (Figure 6A). However in the multivariate Cox regression, association of STAT1 with bad prognosis did not depend on IFN-γ (Figure 6C). Thus, despite the significant association between the expression of IFN-γ and STAT1 transcripts in mammary tumors, the impact on tumor prognosis of these two parameters appears to be non-redundant.

Another intriguing question is the mechanistic link between high *STAT1* and STAT1 target gene expression and bad prognosis. We consider two possibilities, which are not mutually exclusive: First, the transcriptional activation of *STAT1* could lead to expression of one or more critical STAT1 target genes that directly influence tumor progression and metastasis; Second, high STAT1 levels might simply serve as a marker for a chronic inflammatory process which was described to drive the progression and dissemination of the tumor [21,49]. Among the STAT1 targets investigated in our study, *MX1* and *CXCL10* can be considered as genes influencing tumor progression, since their expression was significantly associated with bad patient‘s prognosis (Figure 6). MX1 is described to exert an antiviral activity by binding to cellular RNA helicases required for viral replication but was not ascribed any obvious function in tumor biology [50]. By contrast, two described properties of CXCL10 may underlie its potential tumor-promoting effects: One is its direct action on the proliferation of breast cancer cells [51]. The other is its N-terminal processing by proteases under conditions of chronic infections to a truncated antagonistic CXCL10 form, which impedes chemoattraction of activated lymphocytes and by this means acts as an immunosuppressor [37]. The later mechanism could also be exploited by cells of established, highly inflammatory neoplasms to avoid recognition and killing by tumor-specific T lymphocytes.

In our study, immunohistochemical analysis was able to distinguish the expression of STAT1 in the tumor epithelium and stroma, yet it was not possible to discern whether expression in one of these two compartments was prognostically more relevant (Figure 6A,B). We could identify a correlation of STAT1 mRNA amounts and epithelial STAT1 with markers of infiltrating leukocytes, in particular macrophages (Figure 4A,B and Figure 5). This indicates that either tumor cells with high STAT1 expression are more likely to provide a favorable environment for the recruitment/expansion of macrophages in the tumor or that macrophages promote an environment leading to high *STAT1* expression in the tumor. Whereas the association of STAT1 with infiltrating leukocytes and its impact on bad prognosis in breast cancer is a novel finding of this study, several reports have already documented the impact of infiltrating immune cells, in particular tumor-associated macrophages, on progression of disease and bad outcome [52,53]. By contrast, increased infiltration of the tumor with lymphocytes, in particular T cells, has been associated with better outcome in breast cancer patients subjected to neoadjuvant therapy [54-56]. It remains to be investigated whether the lymphocyte infiltration in the tumor correlates with a better survival in our studied patient collective.

## Conclusions

Our study reveals a complex association between STAT1 activation and progression of breast cancer. STAT1 tyrosine phosphorylation, typically increased after short-term activation of STAT1, is linked to good prognosis for the patient, whereas high levels of STAT1 mRNA, characteristic for sustained activation, predict bad outcome of disease. Furthermore, there was a positive correlation between mRNA levels of STAT1, STAT1 target genes, and marker genes indicative for infiltration with macrophages, pointing to an interrelationship between these parameters. The results of the Cox regression analysis further support a relevant link between STAT1 and macrophage infiltration for explaining bad prognosis.

## Competing interests

The authors declare that they have no competing interests.

## Authors’ contributions

PT designed and performed experiments and contributed to data analysis, interpretation and writing of the manuscript. PC, HH performed statistical data analysis. RS, EM-H and PO carried out the immunohistochemical evaluation of data. FR, JPP and HF contributed patient’s samples, clinical data and participated in the design of the study. ZT participated in the study design. WD conceived and coordinated the study, participated in the immunohistochemical analysis and wrote the manuscript. All authors read and approved the final manuscript.

## Pre-publication history

The pre-publication history for this paper can be accessed here:

http://www.biomedcentral.com/1471-2407/14/257/prepub

## Supplementary Material

Additional file 1: Table S1 No of tumor samples with available data for expression analysis by different methods. Click here for file

Additional file 2: Figure S1 Histograms for STAT1 expression in tumor and stroma. Click here for file

Additional file 3: Table S2 Oligonucleotides for RT-PCR. Click here for file
