# Supplementary material for: High STAT1 mRNA levels but not its tyrosine phosphorylation are associated with macrophage infiltration and bad prognosis in breast cancer
Source: BMC Cancer. 2014 Apr 12;14:257. doi: 10.1186/1471-2407-14-257 (PMC4021106; doi:10.1186/1471-2407-14-257)
Supplement: Additional file 1: Table S1 — No of tumor samples with available data for expression analysis by different methods. [file 1471-2407-14-257-S1.pdf]

Additional file 1 - Supplementary table 1

No of tumor samples with available data for expression analysis by different methods

Type of expression analysis	Cohort A (No. of samples)	Cohort B (No. of samples)
RT-PCR STAT1	96	36
RT-PCR IRF1	96	36
RT-PCR SOCS1	96	0
RT-PCR IFN-γ	92	36
RT-PCR CD68	74	36
RT-PCR CD45	74	0
RT-PCR FOXP3	74	36
RT-PCR CD163	65	21
RT-PCR CXCL9	74	35
RT-PCR CXCL10	74	36
RT-PCR CXCL11	72	34
RT-PCR IFIT	74	36
RT-PCR IFITM1	74	36
RT-PCR MX1	74	36
RT-PCR PD-L1	63	28
RT-PCR PD-L2	73	36
RT-PCR PD-1	47	0
Protein extracts for immunoblotting of total Stat1 and pY701-STAT1	39	0
Protein extracts for immunoblotting of pS727-STAT1	27	0
Protein extracts for CXCL10 ELISA	10	0
Paraffin blocks for STAT1 immunohistochemistry	83	0
