# Supplementary material for: High STAT1 mRNA levels but not its tyrosine phosphorylation are associated with macrophage infiltration and bad prognosis in breast cancer
Source: BMC Cancer. 2014 Apr 12;14:257. doi: 10.1186/1471-2407-14-257 (PMC4021106; doi:10.1186/1471-2407-14-257)

Additional file 2 - Supplementary figure 1

Histograms for STAT1 expression in tumor and stroma

Number of cases with different scores for proportion of stained tumor or stroma cells (0, none; 1, <10%; 2, 10-50%; 3, >50%) as well as the intensity of staining (0, none; 1, weak; 2, moderate; 3, strong) are shown. Scoring was performed for specific expression in nucleus and cytoplasma. A highly similar scoring was obtained by evaluation of only nuclear expression as indicated by the Spearman's correlation coefficients and their two-tailed significances of the two scoring procedures for tumor cells ($r = 0.983$, $p < 0.00001$) and the stroma ($r = 0.823$, $p < 0.00001$).

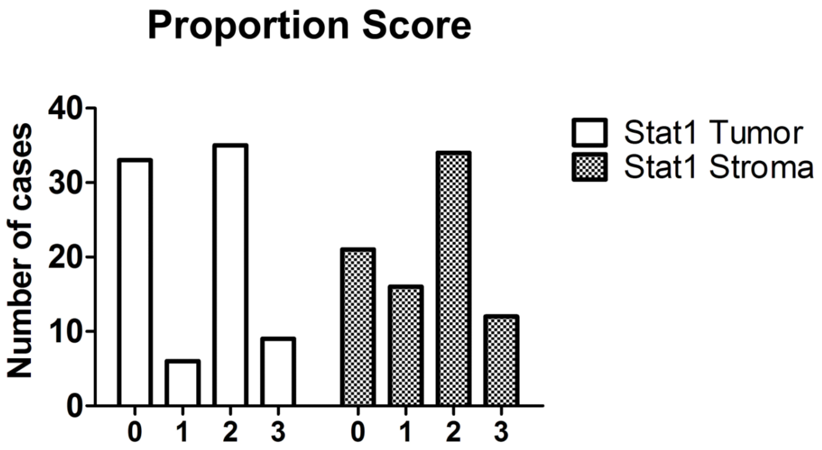

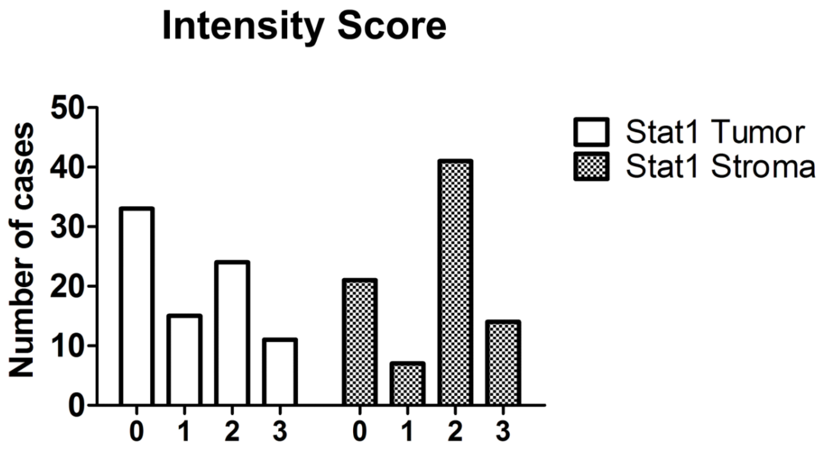

Supplement: Additional file 2: Figure S1 — Histograms for STAT1 expression in tumor and stroma. [file 1471-2407-14-257-S2.pdf]
