# Supplementary material for: High STAT1 mRNA levels but not its tyrosine phosphorylation are associated with macrophage infiltration and bad prognosis in breast cancer
Source: BMC Cancer. 2014 Apr 12;14:257. doi: 10.1186/1471-2407-14-257 (PMC4021106; doi:10.1186/1471-2407-14-257)
Supplement: Additional file 3: Table S2 — Oligonucleotides for RT-PCR. [file 1471-2407-14-257-S3.pdf]

Additional file 3 - Supplementary table 2

Oligonucleotides for RT-PCR

Gene	Accession number [reference]	Method	Forward Primer (5' to 3')	Reverse Primer (5' to 3')	Probe (5' to 3')
TBP	X54993 [1]	TaqMan	CACGAACCACGG CACTGATT	TTTTCTTGCTGCC AGTCTGGAC	TCTTCACTCTTGGC TCCTGTGCACA
STAT1	NM_007315.3	TaqMan	CATTCACATGGG TGGAGCG	GGGTTCAACCGC ATGGAAG	TCCCAGAACGGAG GCGAACCTG
SOCS1	NM_003745.1 [2]	TaqMan	TTTTCGCCCTTAG CGTGAAG	CATCCAGGTGAA AGCGGC	CCTCGGGACCCAC GAGCATCC
IRF1	NM_002198.2	TaqMan	AAGGATGCCTGT TTGTTCCG	CAGCGAAAGTTG GCCTTCC	CTGGGCCATTCACA CAGGCCGATAC
IFN-γ	NM_000619.2	TaqMan	CCAACGCAAAGC AATACATGA	CGCTTCCCTGTTT TAGCTGC	ATCCAAGTGATGG CTGAACTGTCGCC
FOXP3	NM_014009.3	EvaGreen	GGCACAATGTCT CCTCCAGAGA	CAGATGAAGCCT TGGTCAGTGC	N/A[a]
CD68	NM_001251.2	EvaGreen	CGAGCATCATTC TTTCACCAGCT	ATGAGAGGCAGC AAGATGGAC	N/A
CXCL9	NM_002416.1	TaqMan	TTGGGCATCTTG CTGG	GGAACAGCGACC CTTTCTCA	TCTGATTGGAGTGC AAGGAACCCCAGT A
CXCL10	NM_001565.3	TaqMan	TGAAATTATTCC TGCAAGCCAAT	CAGACATCTCTT CTCACCCTTCTTT	TGTCCACGTGTTGA GATCATTGCTACAA TG
CXCL11	NM_005409.4	TaqMan	CCTTGGCTGTGA TATTGTGTGC	CCTATGCAAAGA CAGCGTCCT	CAGTTGTTCAAGGC TTCCCCATGTTCA
IFIT1	NM_001548.4 [3]	EvaGreen	TTGCCTGGATGT ATTACCAC	GCTTCTTGCAAA TGTTCTCC	N/A
IFITM1	NM_003641.3 [3]	EvaGreen	TCTTCTTGAACT GGTGCTGTC	GTCGCGAACCAT CTTCCTGT	N/A
MX1	NM_001144925.1 [3]	EvaGreen	AGGACCATCGGA ATCTTGAC	TCAGGTGGAACA CGAGGTTC	N/A
PD-1	NM_005018.2	EvaGreen	GTGTCACACAAC TGCCCAAC	CTGCCCTTCTCTC TGTCACC	N/A
CD163	NM_004244.5	EvaGreen	TTGCCAGCAGCT TAAATGTG	AGGACAGTGTTT GGGACTGG	N/A
PD-L1	NM_014143.3	EvaGreen	TATGGTGGTGCC GACTACAA	TGCTTGTCCAGA TGACTTCG	N/A
PD-L2	NM_025239.3	EvaGreen	CTGGGTTGGAGC TACTGCAT	GCAATTCCAGGC TCAACATT	N/A

[a]N/A, not applicable

References

1. Bieche I, Onody P, Laurendeau I, Olivi M, Vidaud D, Lidereau R, Vidaud M: **Real-time reverse transcription-PCR assay for future management of ERBB2-based clinical applications**. *Clin Chem* 1999, **45**(8 Pt 1):1148-1156.
2. Haffner MC, Petridou B, Peyrat JP, Revillion F, Muller-Holzner E, Daxenbichler G, Marth C, Doppler W: **Favorable prognostic value of SOCS2 and IGF-I in breast cancer**. *BMC Cancer* 2007, **7**:136.
3. Schild-Hay LJ, Leil TA, Divi RL, Olivero OA, Weston A, Poirier MC: **Tamoxifen induces expression of immune response-related genes in cultured normal human mammary epithelial cells**. *Cancer Res* 2009, **69**(3):1150-1155.
